# Strategies in megasynthase engineering – fatty acid synthases (FAS) as model proteins

**DOI:** 10.3762/bjoc.13.119

**Published:** 2017-06-21

**Authors:** Manuel Fischer, Martin Grininger

**Affiliations:** 1 Institute of Organic Chemistry and Chemical Biology, Buchmann Institute for Molecular Life Sciences, Cluster of Excellence for Macromolecular Complexes, Goethe University Frankfurt, Max-von-Laue-Str. 15, 60438 Frankfurt am Main, Germany

**Keywords:** fatty acid synthases, megasynthases, metabolic enzyme engineering, polyketide synthases, protein design

## Abstract

Megasynthases are large multienzyme proteins that produce a plethora of important natural compounds by catalyzing the successive condensation and modification of precursor units. Within the class of megasynthases, polyketide synthases (PKS) are responsible for the production of a large spectrum of bioactive polyketides (PK), which have frequently found their way into therapeutic applications. Rational engineering approaches have been performed during the last 25 years that seek to employ the “assembly-line synthetic concept” of megasynthases in order to deliver new bioactive compounds. Here, we highlight PKS engineering strategies in the light of the newly emerging structural information on megasynthases, and argue that fatty acid synthases (FAS) are and will be valuable objects for further developing this field.

## Review

### Megasynthases are proteins in natural compound synthesis

**Figure 1:**
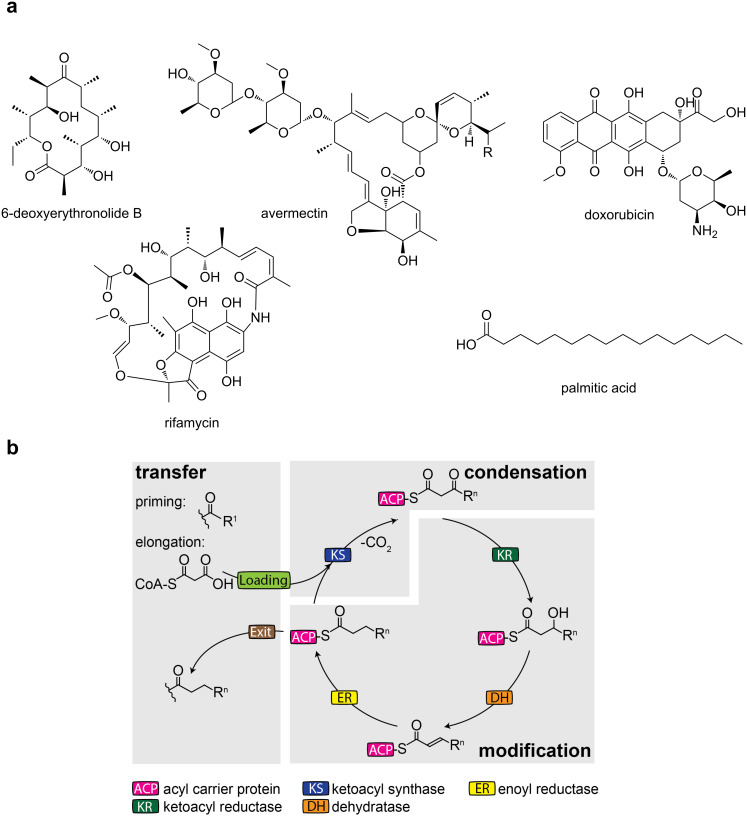
Megasynthases – chemistry and modes of action. a) Products of PKS and FAS megasynthases. b) Reaction cycle of iterative fatty acid biosynthesis as performed by fatty acid synthases (FAS). Synthesis by PKS is essentially similar, except a variation in the degree of β-carbon modification, and the variation in loading and exit transferases. Modular PKS perform one cycle per module before translocating the substrate to the next module.

Microbial natural products represent a rich source of pharmaceutically relevant chemical entities. A major class is represented by polyketides (PK) exemplified by the antibiotics erythromycin and rifamycin, by the antineoplastic doxorubicin and by the antiparasitic avermectin (Figure 1a) [1]. PK are assembled from acyl-coenzyme A (acyl-CoA) units via a series of Claisen-type condensation reactions catalyzed by polyketide synthases (PKS) (Figure 1b). PKS occur as large multifunctional enzymes, termed megasynthases, which harbor the catalytic domains on large polypeptides that can exceed sizes of one MDa [2]. PK compounds are assembled either in a linear manner, where multiple modules successively condense precursor units to the final compound (modular systems) [3], or in a recursive manner, with the catalytic domains of a single module repeatedly condensing precursor units until the specific length/size is attained (iterative systems) [4]. In either case, the enzymatic functions of each module deterministically encode the chemical nature of the final product [5].

### Fatty acid synthases (FAS) are a type of PKS megasynthases

The biosynthetic foundations of PKS are essentially identical to those of FAS. Whereas FAS are strictly fully reducing (Figure 1b), the nature and extent of β-carbon modification varies across the PKS [6]. Though knowledge on PKS has improved in the last decade [7], particularly aided by recent structural studies [8–10], the current insight onto PKS is still significantly built on FAS data. Since the onset of FAS research with the pioneering studies of Bloch, Lynen, Stadtman and Wakil [10–12], FAS have been subject of intense investigation and are today relatively well understood. In recent years, a wealth of structural data on FAS multienzyme complexes (type I) has further deepened the insight into the principles of fatty acid (FA) synthesis [13–19].

### Molecular mechanisms of FAS/PKS mode of action

#### Compartmentalization

**Figure 2:**
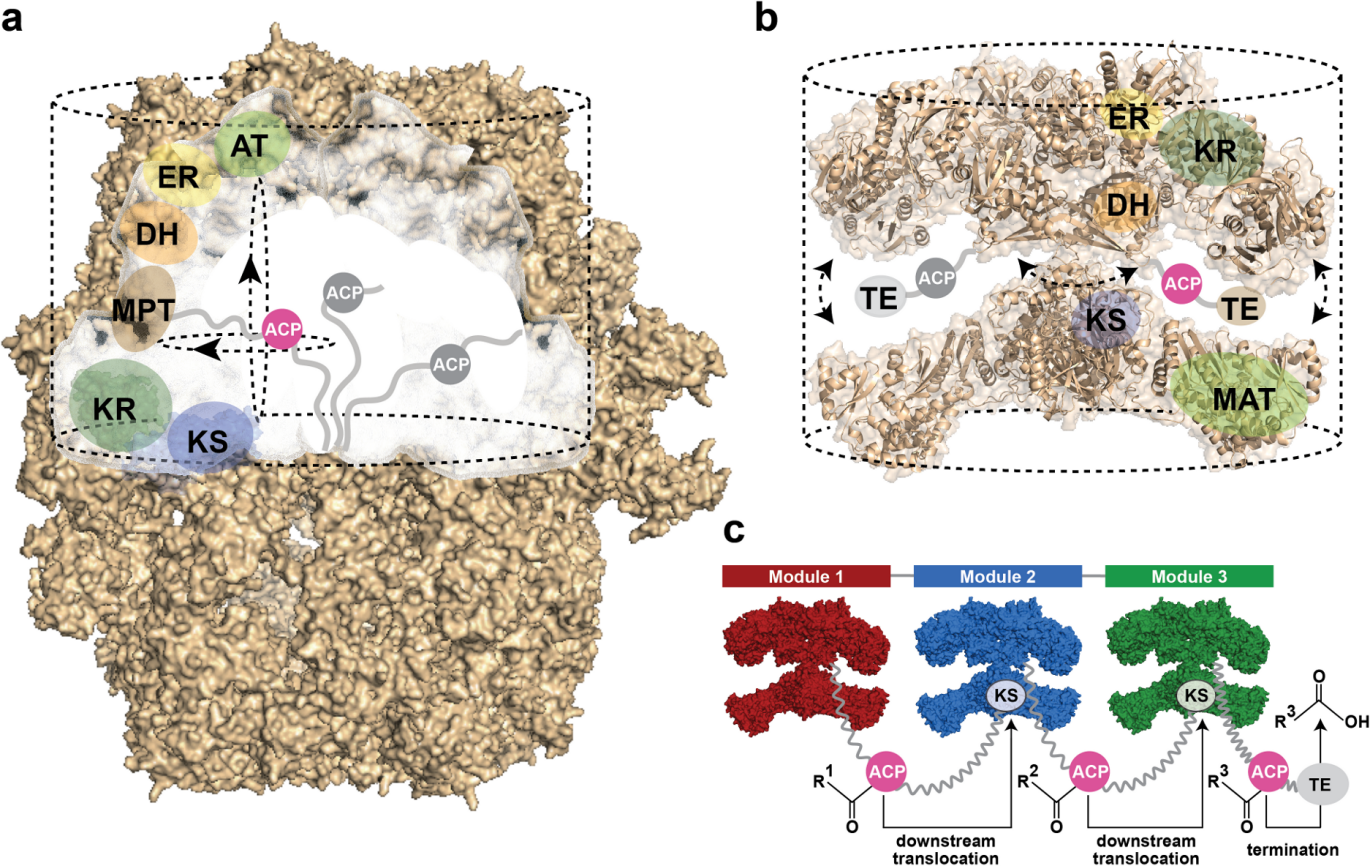
Compartmentalization of synthesis. a) Surface depiction of fungal FAS (PDB-code: 3hmj) with the upper reaction chamber shown without the front part of the barrel wall. One of overall three sets of catalytic domains within a reaction chamber is indicated. Abbreviations as introduced before; additionally, acetyl transferase (AT) and malonyl-/palmitoyl-transferase (MPT). Two linkers, abstracted as grey lines, flexibly bind the acyl carrier protein (ACP) domain. The reaction volume was calculated as indicated by dashed lines. b) Cartoon representation of X-shaped animal FAS I (PDB-code: 2vz8) with one of overall two sets of catalytic domains highlighted. Abbreviations as introduced before; additionally, malonyl-/acetyl-transferase (MAT) and thioesterase (TE). Conformational flexibility of animal FAS, as indicated by arrows, is largely induced by a central waist. The reaction volume was calculated as cylinder as indicated by dashed lines. c) Model of a modular PKS assembly line. Modules 1–3 are depicted in an animal FAS I-like fold. Linkers, covalently or non-covalently connecting modules in modular PKS, are abstracted with zig-zag lines carrying also ACP. Only one of the two ACP domains per homodimeric module is shown for clarity. Each ACP acts *in cis* for substrate elongation (see Figure 2b) and *in trans* for substrate translocation.

Compartmentalization is a phenomenon seen both in FAS as well as PKS systems, but it is differing in its specific structural manifestation. In fungal FAS (and bacterial type I FAS occurring in *Corynebacterium*, *Mycobacterium* and *Nocardia* of the genus *Actinomycetales*), nature evolved a D3-symmetric barrel-shaped structure of 2.6 MDa, which encloses all synthetic processes in two reaction chambers (Figure 2a) [19–20]. The animal FAS exhibits a structurally open homodimeric fold, which shows high conformational flexibility allowing large swinging and swiveling motions (Figure 2b). In animal FAS, synthesis of FA is performed in reaction clefts rather than in enclosed chambers, as found in fungal FAS.

An approximate calculation from the dimensions of the fungal FAS (barrel structure abstracted as cylinder and considering six full sets of active sites per barrel) accounts for a virtual concentration of 1.8 mM of active sites. An analogous consideration for animal FAS (again abstracted as spanning a cylindrical reaction space, two full sets of active sites) gives a virtual active site concentration of 1.2 mM. Accordingly, both scaffolds of FA type I synthesis facilitate reactions at high virtual concentration of enzymatic domains. PKS megasynthases share basic principles with the mammalian FAS fold (Figure 2c) [6–7], and it is valid to assume that active site concentrations lie in the similar range. Bacterial and mitochondrial FA synthesis comprises separate enzymes. To compensate for the lower organizational level, key enzymes occur at copy numbers of about 10,000 (malonyl transferase FabD) to 23,000 (dehydratase FabA), as such being represented within the class of most abundant proteins in *E. coli*; in concentration directly following ribosomal proteins and proteins associated with translation [21]. Calculated with an average volume of an *E. coli* cell of 2.5 µm [3,22], copy numbers account for molar concentrations of about 0.007 to 0.016 mM.

#### Substrate shuttling

FA and PK syntheses generally rely on ACP that shuttles substrates and intermediates as covalently bound cargo between active sites [23]. In FAS and PKS (type I) megasynthases, ACP are embedded as domains in the large polypeptide chains (Figure 2a and b). Held in the compartment, ACP hinders the loss of the covalently attached acyl moiety, realizing high substrate concentrations. In addition to intramodular substrate shuttling, ACP is also responsible for the translocation of the cargo to the downstream modules in modular PKS, which largely accounts for the assembly-line character of these proteins (Figure 2c).

As part of the multienzyme compartment, the mode of ACP action is best described as enabling limited diffusion within a conformational space that is restricted by ACP linkers and the protein scaffold. As calculated from the reported specific activity of 2,500 mU/mg [24], *S. cerevisiae* FAS runs about 18 iterative cycles per second (per set of active sites). Given that each cycle requires six productive interactions between the ACP and the catalytic domains (ACP:KS (ping-step) → ACP:MTP → ACP:KS (pong-step) → ACP:KR → ACP:DH → ACP:ER), *S. cerevisiae* FAS performs a catalytic step every 9.2 milliseconds. This high catalytic efficiency is due to the highly evolutionarily developed architecture of fungal FAS. Enzymatic domains are rigidly embedded into the walls of the reaction chambers, while the ACP domains are held centrally in the chamber by two unstructured linkers of about 20 to 50 amino acid residues in length (40 and 25 amino acids in *S. cerevisiae* FAS). Interestingly, duplicated ACP domains have been observed in certain fungal FAS, and ACP duplication has been ranked as a rather late event during the course of evolution [25]. In the light of the key role of ACP in substrate shuttling, multiple ACP domains might be beneficial in increasing the substrate concentration at which type I synthesis is performed [26–27]. The conformationally more flexible mammalian FAS runs at 2 cycles per second (per set of active sites) calculated from specific activities reported for chicken FAS [28]. Owing to a difficult access to the purified proteins, a limited number of studies report the activity of PKS megasynthases. For example, the modular PKS 6-deoxyerythronolide B synthase (DEBS) shows a turnover number of about 1 min^−1^ over the six elongation steps for product production (accordingly roughly 0.05 elongations per second per set of active sites) [29]. For the iterative PKS 6-methylsalicylic acid synthase (MSAS), a turnover number of about 4.2 min^−1^ over the three iterations for product synthesis was reported (0.1 elongations per second per set of active sites) [30].

#### The function of ACP

The molecular details underlying the ACP mode of action are currently collaboratively decoded via structural, functional and computational methods, disclosing the picture of substrate shuttling being much more than just a mean to keep substrates recruited at the synthetic unit. Most of the understanding about the interaction of ACP with catalytic domains again originates from studies on FAS. Early information was received by *S. cerevisiae* FAS X-ray structures, in which ACP was found in contact with the KS domain [31]. In fungal FAS, ACP is an extended fold comprised of a bacterial-like core fold and a 4-helical extender fold, rendering ACP about twice the size of ACP occurring in mammalian FAS and PKS. The active serine, which is post-translationally phosphopantetheinylated [24,32], is located at the tip of the fold opposite to the N- and C-terminal attachment sites. This structural organization likely preserves linkers from interfering in ACP:domain interactions, and, concomitantly, may support the loading of the covalent acyl moiety by steering the acyl tail into the binding channels. A computational study, on the basis of *S. cerevisiae* FAS data, refined the understanding of ACP-mediated substrate shuttling in *S. cerevisiae* FAS by confirming steering in the sense of promoting correct orientations, as well as suggesting electrostatic steering by charge complementarity of the surfaces of binding partners [33]. Recent studies have characterized ACP of FAS megasynthases as not sequestering the covalently bound acyl moiety, which is supportive of molecular steering effects underlying substrate shuttling [34–35]. Specific structural information on the interaction of ACP with the catalytic domains is otherwise rare, hindered by the transient nature of this event. The application of specific crosslinkers aided in overcoming this difficulty for the interaction of ACP with the FAS type II dehydratase FabA [36–37]. This study was the first in tracing key events in ACP docking and acyl-moiety binding, and allowed catching an initial glimpse of the dynamic process of ACP substrate delivery. Also the interaction of ACP VinL and the acyltransferase VinK, involved in loading a PKS megasynthase, was recently resolved in structure [38]. It is reasonable to assume that the ACP mode of action in PKS is similar to FAS. The role of ACP in modular PKS is, however, complicated by the additional task of delivering the acyl moieties also to the downstream module (Figure 2c). Just rudimentary information on the nature of this translocation step is available; most importantly suggesting ACP to dock with different faces during intra- and intermodular acyl-chain delivery [39–40].

### Strategies for megasynthase engineering

The concept of one multienzyme module being responsible for the incorporation of one building block in modular systems has inspired chemists and biologists for more than two decades to create engineered pipelines for the directed synthesis of bioactive compounds [41–42]. Engineering of megasynthases provides the opportunity to complement or replace synthetic chemical strategies for natural compound production with sustainable, green-chemistry approaches. Several reports on the engineering of PKS have proven the feasibility of the concept [43–44], but megasynthase design as a tool for the custom synthesis of natural compounds or complex precursor molecules has remained elusive to date [45–46].

**Figure 3:**
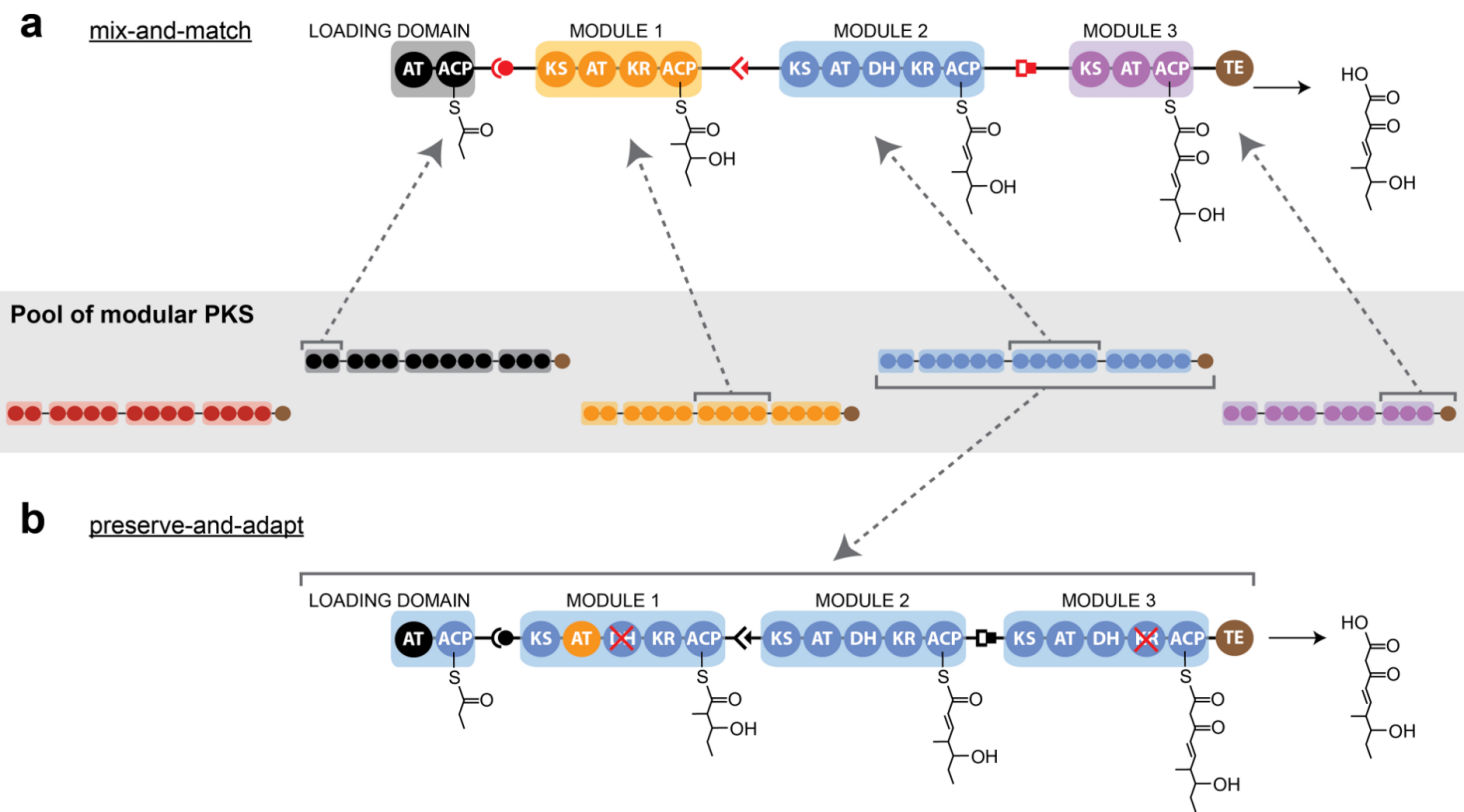
Strategies of megasynthase engineering. a) Mix-and-match approach: A hypothetical chimeric PKS is assembled module by module from a pool of available PKS. In such approach, docking domains (represented in red color) are adapted for coding the sequence of assembly. b) Preserve-and-adapt approach: A modular PKS, selected from the native pool by beneficial properties (see text) is used as a scaffold and adapted in catalytic functions and substrate specificities.

Towards the desirable goal to produce PK by designing the respective megasynthase, the chemical/biological community has largely performed the approach of assembling modules and domains from interchangeable units. Mainly by addition, removal and/or substitution of modules and domains (Figure 3a), libraries of compounds have been generated with varying patterns of functional groups [1,47–50]. In the light of the emerging knowledge on the complex role of ACP during intramodular and intermodular interactions, as well as the yet essentially unclear principles of module–module interactions [8,51–52], the idea of an unhindered vectorial transport through such chimeric assembly line PKS may seem naive. Clearly, research over the last two decades has demonstrated that modules and domains are not interchangeable per se [45–46], and a successful mixing-and-matching approach will significantly depend on engineering clashing interfaces.

An alternative approach towards harnessing PKS for custom-product synthesis, may be built on the concept of establishing selected PKS as generic scaffolds (“chassis”). In such an engineering strategy, a PKS scaffold is first selected and then adapted to the requirement of a specific synthesis; the latter essentially requiring the engineering of active sites and binding channels for accepting and processing desired substrates and intermediates (Figure 3b). In using a related terminology as for the approach of domain and module recombination (“mix-and-match”), such an alternative approach may be termed “preserve-and-adapt”. While the adaptation of active sites will remain as a challenging task in such an approach, the generally profound description of substrate/active site complexes, the conservation of active sites beyond protein families, and their susceptibility for biophysical assays makes the engineering of substrate specificities a promising alternative to the mix-and-match approach; particularly as domain–domain and module–module interactions are comparably difficult to engineer. The benefit of a preserve-and-adapt approach lies in the non-invasive nature to the overall structural frame of an assembly line, i.e., keeping module–module and domain–domain interactions as well as substrate shuttling intact. Another advantage is that such PKS scaffolds could be selected for suited properties, as, e.g., expression levels and protein quality in recombinant hosts; likely an aspect, which is underestimated in mix-and-match approaches. A preserve-and-adapt approach might moreover be aided by the evolutionary loosely developed substrate specificity of megasynthases. As megasynthase-mediated synthesis is subject to substrate shuttling, achieving high local concentrations of substrates and mediating specificity of the system via domain–domain interactions, evolution has likely not selected for strict substrate specificity as compared to diffusion-loaded proteins, and megasynthases might be inherently substrate tolerant [53–55].

### Preserve-and-adapt approach on the example of FAS

**Figure 4:**
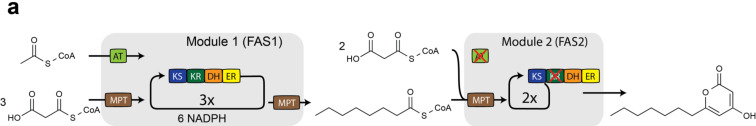
Preserve-and-adapt approach with FAS. *C. ammoniagenes* FAS has been engineered in two cooperatively acting modules to produce 6-heptyl-4-hydroxypyran-2-one. Module 1, mutated in domains KS and MPT, is responsible for the release of the acyl ester C_8_-CoA, and module 2 for the non-reductive elongation of C_8_-CoA, induced by a KR-domain functional knockout, to synthesize the final lactone PK. The knockout in the AT-domain hinders the uptake of acetyl-CoA and encodes for modules acting in sequence.

Given the detailed understanding of their structural and functional properties, FAS are ideal proteins for evaluating a preserve-and-adapt engineering strategy on megasynthases in an in vitro environment. We therefore recently started the specific project of installing the synthesis of short-chain FA (SCFA) and the polyketide lactone 6-heptyl-4-hydroxypyran-2-one (6-HHP) within the scaffold of the *Corynebacterium ammoniagenes* FAS (a bacterial type I system). In an engineered reaction sequence, an initial FAS module was designed to produce SCFA as acyl esters, which are in a second FAS module elongated to the triketide and cyclized to the final lactone (Figure 4). A similar synthetic route can be found in norsolorinic acid synthesis, in which a fully reducing fungal FAS collaborates with a non-reducing PKS [4,56], as well as in resorcylic acid lactone synthesis, in which two iterative PKS systems work in sequence [57]. We selected this reaction route, as it involves the engineering of the condensation domain KS and the transferase domains AT and MPT that make up the catalytic core in PKS/FAS proteins. This approach was successful in finally obtaining the desired compound in 35% yield by overall just implementing five mutations [58].

When introducing module 1 mutations into baker’s yeast, the technologically relevant SCFA were produced by C_8_-CoA being hydrolyzed and exported to the culture medium [59]. Particularly in this function, module 1 is interesting for comparing the preserve-and-adapt approach with other strategies employed to date for producing SCFA. By adapting active site specificities, mutations essentially steer de novo fatty acid synthesis towards the early release of not yet fully elongated C_16_ and C_18_-acyl-CoA, while leaving the overall molecular mechanisms intact. Indeed, evaluated on the basis of SCFA yields, the approach turned out to be highly powerful compared to other strategies that were overwriting native synthesis with a short-chain acyl-ACP specific thioesterase that is inserted as extra domain into the polypeptide chain [60–63].

Further studies on FAS can be envisioned, i.e., when considering the PKS-like mammalian FAS fold. Already at this level, the proof-of-concept performed on FAS can, however, serve as a seed for starting efforts in also making PKS amenable to de novo pathway design; being well aware that in-depth characterization of PKS with enzymological techniques is further needed to collect quantitative data that can inform rational engineering efforts.

## Conclusion

Transient and static domain–domain and module–module interactions as part of an “assembly-line synthetic concept” are still poorly understood. The emerging picture from a fast growing knowledge about the structure and function of megasynthases suggests an impact of these interfaces in megasynthase-mediated natural compound synthesis that can hardly be overstated. The limited success rate of mix-and-match engineering experiments, programming the assembly of domains and modules to new megasynthases, may well be traced back to weakly cooperating domains and modules in these chimeric systems. We suggest that preserve-and-adapt approaches are valuable alternative strategies in rational megasynthase design. Instead of mixing and matching modules, a preserve-and-adapt approach is based on the intact native megasynthase scaffold, in which overall structural properties remain preserved, while the individual active sites are adapted for embedding custom syntheses.
